# Surface Modification of Polyurethane Membrane with Various Hydrophilic Monomers and N-Halamine: Surface Characterization and Antimicrobial Properties Evaluation

**DOI:** 10.3390/polym13142321

**Published:** 2021-07-15

**Authors:** Chi-Hui Cheng, Han-Cheng Liu, Jui-Che Lin

**Affiliations:** 1 Department of Pediatrics, College of Medicine, Chang Gung University, aoyuan 33305, Taiwan; pedneph.cheng@msa.hinet.net; 2 Department of Pediatrics, Chang Gung Memorial Hospital, Taoyuan 33305, Taiwan; 3 Department of Chemical Engineering, National Cheng Kung University, Tainan 70101, Taiwan; apple4030312@gmail.com; 4 Institute of Oral Medicine, College of Medicine, National Cheng Kung University, Tainan 70101, Taiwan

**Keywords:** polyurethane, surface modification, N-halamine, hydrophilic monomer, antimicrobial properties

## Abstract

Reducing microbial infections associated with biomedical devices or articles/furniture noted in a hospital or outpatient clinic remains a great challenge to researchers. Due to its stability and low toxicity, the N-halamine compound has been proposed as a potential antimicrobial agent. It can be incorporated into or blended with the FDA-approved biomaterials. Surface grafting or coating of N-halamine was also reported. Nevertheless, the hydrophobic nature associated with its chemical configuration may affect the microbial interactions with the chlorinated N-halamine-containing substrate. In this study, a polymerizable N-halamine compound was synthesized and grafted onto a polyurethane surface via a surface-initiated atom transfer radical polymerization (SI-ATRP) scheme. Further, using the sequential SI-ATRP reaction method, different hydrophilic monomers, namely poly (ethylene glycol) methacrylate (PEGMA), hydroxyethyl methacrylate (HEMA), and [2-(methacryloyloxy) ethyl] dimethyl-(3-sulfopropyl) ammonium hydroxide (SBMA), were also grafted onto the polyurethane (PU) substrate before the N-halamine grafting reaction to change the surface properties of the N-halamine-modified substrate. It was noted that the chains containing the hydrophilic monomer and the polymerizable N-halamine compound were successfully grafted onto the PU substrate. The degree of chlorination was improved with the introduction of a hydrophilic monomer, except the HEMA. All of these hydrophilic monomer-containing N-halamine-modified PU substrates demonstrated a more than 2 log CFU reduction after microbial incubation. In contrast, the surface modified with N-halamine only exhibited significantly less antimicrobial efficacy instead. This is likely due to the synergistic effects caused by the reduced chlorine content, as well as the reduced surface interactions with the microbes.

## 1. Introduction

Healthcare-associated infections (HAIs) are bacterial infections suffered by hospitalized patients or those receiving medical treatment in an outpatient setting. In recent years, due to the emergence of drug-resistant bacteria, such as methicillin-resistant Staphylococcus aureus, MRSA, HAIs increase significantly annually. The reasons for this phenomenon can be inferred from the fact that bacteria are transmitted from the hospital by the medical staff or patients themselves through the seats in the hospital, surgical clothes, or gloves, to name a few. These surfaces are susceptible to unspecific microbial adhesion/adsorption to form biofilms, which makes them even more difficult to be removed by regular sanitization methods. Therefore, utilizing materials with good antimicrobial activity is essential to effectively interrupt the transmission of pathogens, reduce microbial cross-contamination, and even more, prevent the occurrence of multiple drug resistant (MDR) microbial strains to promote public health [1,2,3,4].

Among the antimicrobial agents, extensive research on N-halamine has been reported in recent years due to its advantages of long-term stability, low toxicity to humans, superior antimicrobial efficacy against broad-spectrum microbes, and special rechargeable properties upon exposure to household bleach [5,6]. Different approaches, including copolymerization, grafting, blending, and coating, have been used to combine N-halamine with conventional polymers to achieve antimicrobial functions [7,8,9,10,11,12]. However, since the chemical structure of N-halamine is intrinsically hydrophobic, substrates modified with N-halamine usually present high hydrophobicity which could lead to less optimum antimicrobial efficacy than expected.

Polyurethane has been widely used in various biomedical applications due to its biocompatibility, low cost, high mechanical elasticity, and chemical stability. Additionally, the polyurethane surface can be modified to improve its biocompatibility [13,14,15]. In this study, to confer proper antimicrobial properties, an N-halamine compound with a vinyl structure, 3-(4’-vinylbenzyl)-5,5-dimethylhydantoin (VBDMH), was surface grafted onto the polyurethane substrate using the surface-initiated atom transfer radical polymerization (SI-ATRP) technique. Further, a sequential SI-ATRP reaction scheme was utilized to incorporate different hydrophilic monomers, namely poly (ethylene glycol) methacrylate (PEGMA), hydroxyethyl methacrylate (HEMA), and zwitterionic [2-(methacryloyloxy) ethyl] dimethyl-(3-sulfopropyl) ammonium hydroxide (SBMA) monomer with the VBDMH to endow the N-calamine modified PU substrate with different hydrophilicity. The surface characteristics and antimicrobial capability of these N-halamine modified PU substrates were examined. To the best of our knowledge, the utilization of hydrophilic monomers with the N-halamine compound for the preparation of antimicrobial substrates has not been reported so far. Results have indicated that, besides changing the surface characteristics, the incorporation of hydrophilic monomers could further enhance the antimicrobial capability of the N-halamine modified PU substrate.

## 2. Materials and Methods

### 2.1. Materials

5,5-dimethylhydantoin (DMH), 4-vinylbenzyl chloride, copper(I) bromide, [2-(Methacryloyloxy) ethyl] dimethyl-(3-sulfopropyl) ammonium hydroxide (SBMA), poly (ethyl glycol) methacrylate (PEGMA, M_n_ = 360 Da), and ethyl-2-bromoisobutyrate (EBIB) were purchased from Sigma-Aldrich (Sigma-Aldrich, St. Louis, MO, USA). 2-bromoisobutyryl bromide (BIBB), 2-(Dimethylamino) pyridine, 2-2’-bipyridine (bpy), and 2-hydroxyethyl methacrylate (HEMA) were purchased from Alfa Aesar (Lancashire, UK). Triethylamine and ethanol were purchased from J.T. Baker (Allentown, PA, USA). Methanol, dichloromethane, and toluene were from MACRON (Allentown, PA, USA). N,N-dimethylformamide (DMF) was acquired from Duksan (Ansan, Korea). All the above chemicals were purchased at the highest purity grade. The thermoplastic ester-based polyurethane pellet (TPU, Estane, S385A) was purchased from Lubrizol Advanced Materials, (Inc., Wickliffe, OH, USA).

### 2.2. Synthesis of 3-(4’-Vinylbenzyl)-5,5-dimethylhydantoin (VBDMH)

**Scheme 1:**
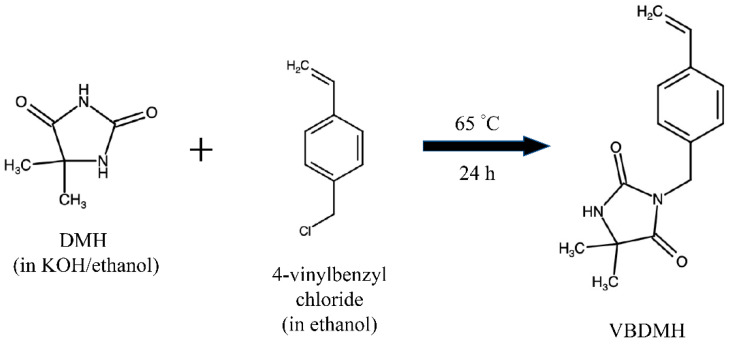
Synthesis of VBDMH.

25.6 g (0.20 mol) of DMH and 11.2 g (0.20 mol) of KOH were dissolved in 120 mL ethanol at 90 °C under constant stirring for 1 h. A solution of 30.8 g (0.2 mol) of 4-vinylbenzyl chloride in 40 mL methanol was added into the previous solution in a dropwise manner. The mixture was stirred at 65 °C overnight. After removing the solvent under reduced pressure at 40 °C, the solid was collected and recrystallized from methanol/water (5:1 *v*/*v*). Then, the resulting solid was washed several times with water and dichloromethane to remove excess salts [16,17]. VBDMH was obtained as white powders after removing the solvent in a vacuum oven overnight and stored under a vacuum before use (Scheme 1).

### 2.3. Surface Modification of PU Membranes

#### 2.3.1. Preparation of PU Membranes

To prepare the PU membranes, the TPU solution (9.5 wt% in dimethylformamide (DMF)) was poured into a round-shape polytetrafluoroethylene (PTFE) mold (ID = 7.5 cm). The TPU solution was then degassed under a vacuum oven overnight, and then the solvent was evaporated under a vacuum oven at 40 °C. The resulting membranes were cut into a square (1 × 1 cm^2^) and were cleaned with methanol for 24 h by Soxhlet extraction and dried under a vacuum oven for further use.

#### 2.3.2. Preparation of Initiator Immobilized PU Membranes (PU-Br)

The PU substrate’s surface was first activated by atmospheric pressure plasma. The treated substrates were kept in the air for few minutes before transforming into an argon-protected three-neck flask.

**Scheme 2:**
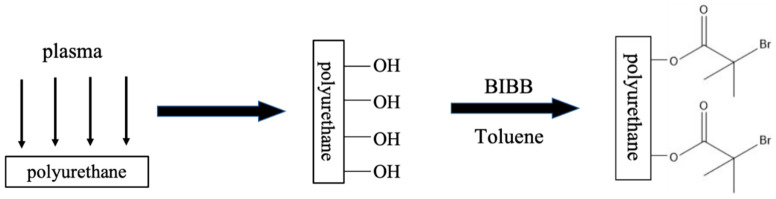
Initiator immobilization on PU membrane (PU-Br).

132 mmol of anhydrous triethylamine and a few drops of 2-(dimethylamino) pyridine were added in the three-neck flask that contained 150 mL toluene under argon protection in an ice bath for 30 min. The initiator of 2-bromoisobutyryl bromide (BIBB, 60 mmol) in 30 mL toluene was injected into the funnel that was pre-equipped on the three-neck flask. The initiator solution was dropped slowly for about 30 min and the mixture reacted for 24 h at room temperature. After the completion of the reaction, the PU substrates were removed from the flask, extracted with methanol to remove the impurities, and dried under a vacuum overnight. The initiator-immobilized PU substrates here are referred to as PU-Br (Scheme 2).

#### 2.3.3. Surface Grafting Polymeric Chains with Surface-Initiated Atom Transfer Radical Polymerization (SI-ATRP)

**Scheme 3:**
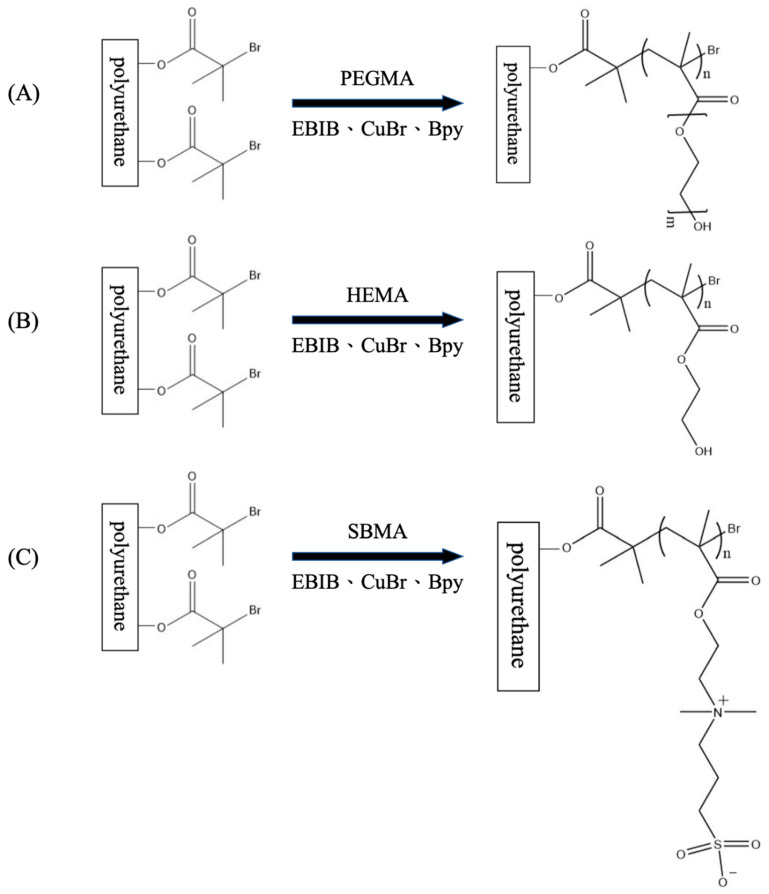
Surface grafting of hydrophilic polymeric chains. (**A**) PEGMA; (**B**) HEMA; (**C**) SBMA.

The hydrophilic polymeric chains were grafted onto the PU-Br substrates by the SI-ATRP technique as shown in Scheme 3. Three different hydrophilic monomers (PEGMA, HEMA, SBMA, 10 mmol), 2-2’-bipyridine (0.2 mmol), and ethyl-2-bromoisobutyrate (0.1 mmol) were dissolved in the solvent and purged with argon for 30 min. Cu(I)Br (0.1 mmol) and PU-Br substrates were placed in the Schlenk tube, after which the tube was vacuumed and backfilled with argon three times. Then, the mixture was transferred to the Schlenk tube, freeze-pump-thawed thrice, back-filled with argon, and the polymerization was conducted at room temperature for 24 h. After the completion of the polymerization, the PU substrate was thoroughly cleaned in methanol and dried in a vacuum oven [18,19,20,21].

**Scheme 4:**
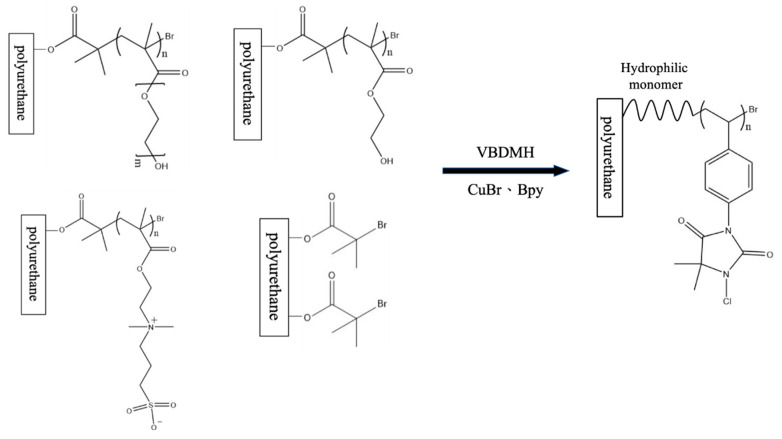
Surface grafting of VBDMH polymeric chains onto different PU substrates.

The VBDMH was then grafted onto the PU substrates, grafted with or without the hydrophilic chains by the SI-ATRP method (Scheme 4). VBDMH (1.22 g, 5 mmol) and 2-2’-bipyridine (0.2 mmol) were dissolved in 8 mL methanol and purged with argon for 30 min. Cu(I)Br (0.1 mmol) and the treated substrate were placed in the Schlenk tube, after which the tube was vacuumed and backfilled with argon three times. The mixture was transferred to the tube, freeze-pump-thawed thrice, back-filled with argon, and the polymerization was conducted at room temperature for 24 h. After the completion of the polymerization, the PU substrate was thoroughly cleaned in methanol and dried in a vacuum oven.

These surface hydrophilic monomer-modified PU substrates were named polyPEGMA, polyHEMA, and polySBMA, with the respective hydrophilic monomers. And the samples obtained after the following VBDMH surface grafting reaction were termed as polyPEGMA/polyVBDMH, polyHEMA/polyVBDMH, and polySBMA/VBDMH, respectively. For the PU substrate without the hydrophilic surface chain grafting, the sample was named polyVBDMH after the VBDMH SI-ATRP grafting reaction.

### 2.4. Chlorination and Titration

The N-halamine-modified PU substrates, the ones grafted with VBDMH polymeric chains, were chlorinated by soaking in a 10% diluted aqueous bleach solution (Chlorox^®^) with HCl to adjust the pH value to 7.0 for 1 h. After chlorination, the surface of the substrates was washed thoroughly with deionized water and dried in a 50 °C oven for 1 h to remove free chlorine. The pristine PU substrates were also treated with the same conditions to serve as a control. The presence of oxidative chlorines (Cl^+^) was determined using the iodometric/thiosulfate titration method [22,23]. The PU substrate after chlorination was put in a flask with 20 mL of deionized water, 1 mL of 0.1 N of acetic acid, and an amount of KI and stirred at room temperature for 1 h to form I_2_. Then, a few drops of the starch indicator were added into the mixture and titrated by 0.5 μN of sodium thiosulfate aqueous solution from blue to colorless. The Cl^+^ (μg/cm^2^) was calculated according to the following equation:

Cl^+^ (μg/cm^2^) = (35.45 × N × V)/(2 × A) (1)

where Cl^+^ (μg/cm^2^) is the amount of oxidative chlorine on the sample surface, N and V are the normality (equiv/L) and volume (mL) of the titrant sodium thiosulfate, respectively, and A (cm^2^) is the surface area of substrates.

### 2.5. Characterization Methods

The chemical structure of VBDMH was confirmed by a nuclear magnetic resonance spectrometer (NMR, AV-500, BRUKER, 500 MHz, Rheinstetten, Germany). The deuterated DMSO was added as a solvent.

The surface chemical bonding and atomic composition were characterized by attenuated total reflectance Fourier transform infrared spectroscopy (ATR-FTIR, Varian 640-IR, Santa Clara, CA, USA) and X-ray photoelectron spectroscopy (XPS, PHI Quantera II, ULVAC-PHI, Inc. Kanagawa, Japan). The ATR-FTIR spectra were acquired after 64 scans with a resolution of 4 cm^−1^. The X-ray source for the XPS measurement was the monochromatic Al-Kα (hν = 1486.6 eV, step size = 0.1 eV, pass energy = 55 eV) with the take-off angle at 45°. The high-resolution spectra were deconvoluted by mixing the Gaussian–Lorentzian functions using the free software program, XPSPEAK. Quantification of the element was performed on the peak areas with the consideration of sensitivity factors of each element provided by the instrument maker.

To determine the surface hydrophilicity of various modified and pristine PU substrates, static water contact angle measurements (WCA; Model 100SB, Sindatek, Taipei, Taiwan) were performed at room temperature (25 °C) using the sessile drop method with deionized water droplets.

### 2.6. Antimicrobial Activity Testing

The bacteria examined were *E. coli* (ATCC 23501) and *S. aureus* (ATCC 21351). The antimicrobial activity was determined according to procedures reported in the Japanese Industrial Standard JIS Z 2801: 2000 (E) [24]. The surface of the test specimen was sterilized with 75% ethanol twice, dried, and further sterilized with UV for 10 min before testing. Then, the untreated and treated samples (1 cm × 1 cm) were placed in a sterilized petri dish with the intended-to-be-examined surface facing upward. The bacteria suspension with 1.0~4.0 × 10^5^ colony forming units (CFU) was placed on the tested specimen surface. The surface was then covered with a sterilized cover glass to ensure the bacterial suspension inoculum was uniformly spread out while not spilling over from the edge of the film. The samples were then incubated at 37 °C at a relative humidity of 90% for 24 h. After incubation, the samples and cover glass were placed within a sterilized pouch and then rinsed with 5 mL SCDLP 80 broth (Soybean Casein Digest Lecithin Polysorbate 80 Medium) [24] and 5 mL 0.001 N sodium thiosulfate solution in a sterilized stomacher with a vortex for 3 min. To quantify the viable bacteria on the samples [24], the bacteria in the washing solution were diluted to a proper concentration, spread on the agar plate, and incubated at 37 °C for 24 h. The number of viable bacteria in the suspensions was determined as CFU. Further, the SEM analyses were performed to ensure the successful removal of the adhered bacteria from the tested specimen and cover glass. The antimicrobial activity, R, was determined as follows [24].

(2) $$R=\left[\log\left(\frac{B}{A}\right)-\log\left(\frac{C}{A}\right)\right]$$

## 3. Results and Discussion

### 3.1. VBDMH Characterization

**Figure 1:**
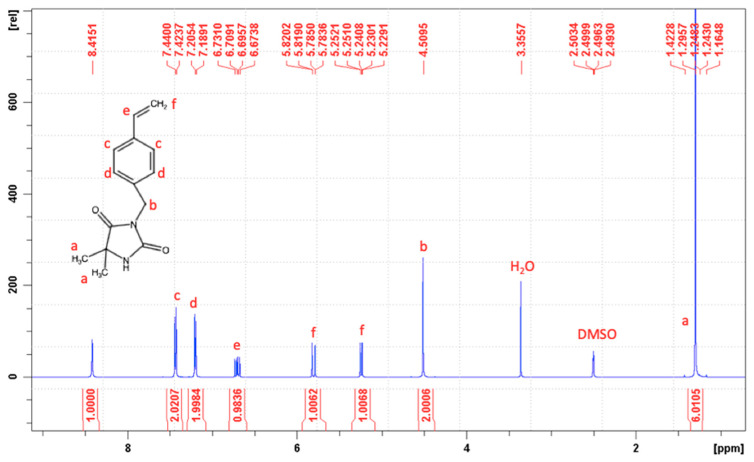
The ^1^H-NMR spectrum of the VBDMH synthesized.

The ^1^H-NMR spectrum of VBDMH synthesized was shown in Figure 1. The chemical shifts in 1H-NMR (500 MHz, DMSO-*d_6_*) δ (ppm) were 1.16–1.42 (6H, –C(CH_3_)_2_–); 4.51 (2H, –N–CH_2_–C–); 5.22–5.25, 5.78–5.82 (2H, –CH=CH_2_–); 6.67–6.73 (1H, –CH=CH_2_–); and 7.18–7.20, 7.42–7.44 (4H, phenyl). This indicated the success of VBDMH synthesis.

### 3.2. Surface Characterization of Different Modified PU Substrates

#### 3.2.1. Surface Hydrophilicity

**Figure 2:**
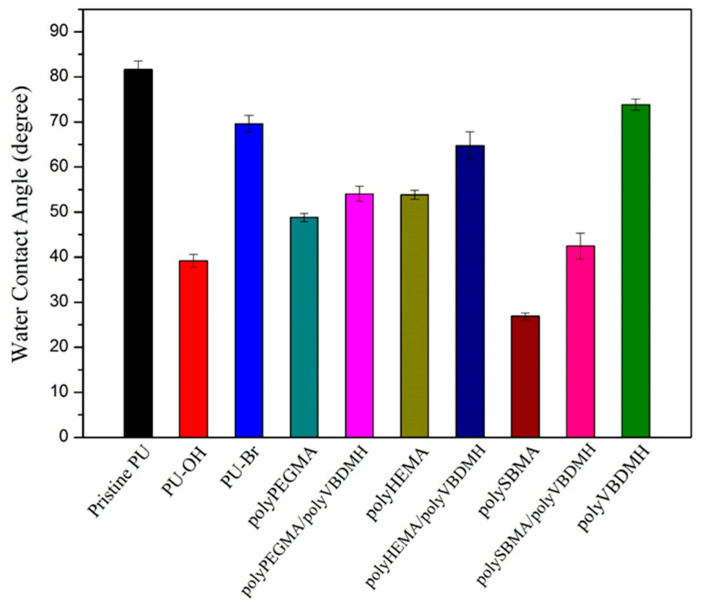
The contact angle value of different PU specimens (n = 3).

The contact angle values of the different specimens are shown in Figure 2. After the plasma torch activation reaction, the substrate (PU-OH) became hydrophilic, resulting from the incorporation of –OH functionalities onto the PU substrate surface. Following the initiator immobilization, the substrate (PU-Br) turned to be hydrophobic with a contact angle of 69.5° ± 1.9°.

After the first SI-ATRP grafting reaction with three different hydrophilic monomers, namely PEGMA, HEMA, and SBMA, the substrates became hydrophilic (contact angle for polyPEGMA: 48.8° ± 0.9°; polyHEMA: 53.8° ± 1.0°; polySBMA: 26.9° ± 0.7°). The one grafted with SBMA showed the lowest contact angle value, and this likely results from its zwitterionic nature that leads to easier water adsorption [25,26]. Following the VBDMH grafting reaction, the contact angle values of all these three hydrophilic monomer grafted substrates increased (polyPEGMA/polyVBDMH: 56.0° ± 1.7°; polyHEMA/polyVBDMH: 64.7° ± 3.1°; polySBMA/polyVBDMH: 42.5° ± 2.9°). In contrast, the polyVBDMH substrate, the direct VBDMH grafted PU substrate that was not first modified with the hydrophilic grafting reaction, showed an even higher hydrophobicity (73.9° ± 1.2°). This indicated the hydrophilic segments incorporated in the first SI-ATRP reaction could reduce the water contact value.

#### 3.2.2. ATR-FTIR Analysis

**Figure 3:**
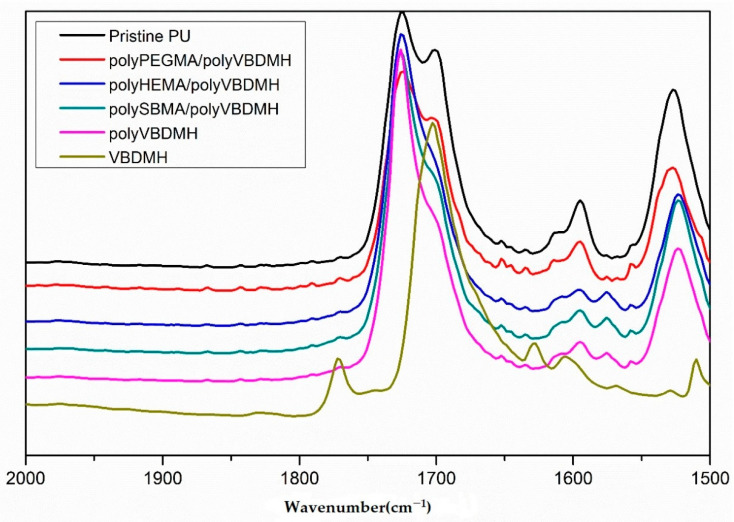
The ATR-FTIR spectra of different PU substrates and VBDMH monomer.

The ATR-FTIR spectra of PU substrates grafted with four different VBDMH-containing chains, the pristine PU substrate, and the VBDMH monomer were shown in Figure 3. The VBDMH monomer exhibited peaks centered at about 1702.8 cm^−1^ and 1770.3 cm^−1^ which can be assigned to the amide carbonyl and imide linkage [16]. Nevertheless, these two peaks became less distinctive after the VBDMH chains being grafted onto the PU substrate (i.e., polyVBDMH). This can be attributed to the overlapping of the adsorption peaks between the VBDMH monomer and pristine PU substrate. Similar findings were also noted on the three PU substrates which were grafted with hydrophilic segments before the VBDMH.

#### 3.2.3. XPS Analysis

XPS, a more surface-sensitive analysis technique than ATR-FTIR, was further utilized to explore the variations in the surface chemical configuration of different modified PU substrates.

The increase of O/C after plasma torch treatment indicated the incorporation of hydroxyl functionalities on the PU-OH substrate (Table 1). This was further supported by the curve-fitting results, for which the area percentage in the peak centered at 286.5 eV, which can be attributed to the C–O–C/C–O/C–N functionalities [18,20,27,28], was increased on the PU-OH surface (Table 2). Besides the increase in O/C ratio, the Br atoms were also noted on the BIBB initiator-immobilized PU membranes (PU-Br) (Table 1), implicating the success in the surface immobilization of the BIBB initiator.

After the first SI-ATRP grafting of different hydrophilic monomers, the surface atomic percentage values of the modified PU substrates varied differently. For the PU substrate grafted with PEGMA (i.e. polyPEGMA) and HEMA (polyHEMA), the N1s percentage decreased, while the C1s and O1s remained similar as compared to the PU-Br (Table 1). This implicated the formation of a layer of grafting polymers on the PU substrate. Nevertheless, for the one modified with SBMA (polySBMA), due to SBMA chemical configuration, the N1s did not vary significantly, while the S2p signal was noted (Table 1). However, the notion in Br atoms on the polySBMA implicated the grafting polymeric chains may not be thick enough as compared to another two substrates, polyPEGMA and polyHEMA, to block the Br photoelectrons reaching the XPS detector. After further SI-ATRP grafting with VBDMH, it was speculated that the thickness increase in the grafting polymer layer has resulted in the decrease in Br3d surface atomic percentage in polySBMA/polyVBDMH (Table 1). For the polyPEGMA/polyVBDMH and polyHEMA/polyVBDMH modified substrates, a significant increase in N1s atomic percentage than its counterpart, polyPEGMA, and polyHEMA, suggested the incorporation of polymerized VBDMH grafting chain after the 2nd SI-ATRP reaction (Table 1).

**Figure 4:**
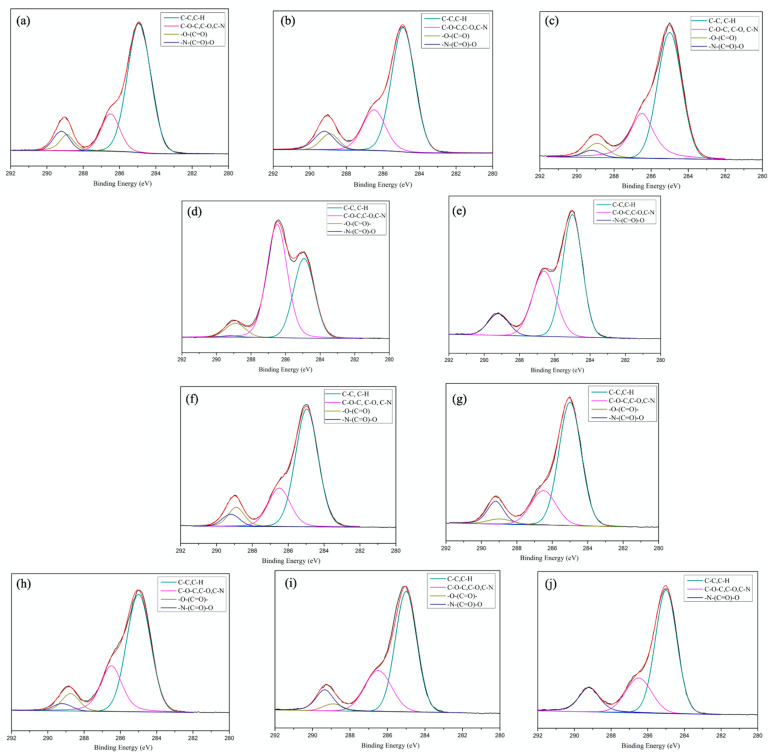
The C1s spectra of different PU specimens: (**a**) pristine PU; (**b**) PU-OH; (**c**) PU-Br; (**d**) polyPEGMA; (**e**) polyPEGMA/polyVBDMH; (**f**) polyHEMA; (**g**) polyHEMA/polyVBDMH; (**h**) polySBMA; (**i**) polySBMA/VBDMH; (**j**) polyVBDMH.

The C1s spectrum and the C1s curve fitting results of different PU substrates are shown in Figure 4 and Table 2. It was noted that the surface chemical composition would vary to a different extent after different surface treatment modalities, as well as the monomer used. After the second SI-ATRP grafting with VBDMH, all three substrates, polyPEGMA/polyVBDMH, polyHEMA/polyVBDMH, and polySBMA/polyVBDMH, demonstrated a significantly higher area percentage in –N–(C=O)–O (289.2 eV) than its counterpart that was only grafted with a layer of hydrophilic monomer (Table 2). As compared to the curve fitting results for the PU substrate “only” modified with VBDMH chains (i.e. poyVBDMH), these findings can further indicate the success in the surface grafting of polymerized VBDMH chains following the previous grafting with three hydrophilic monomers.

### 3.3. Chlorination of Different VBDMH Modified PU Substrates 

**Figure 5:**
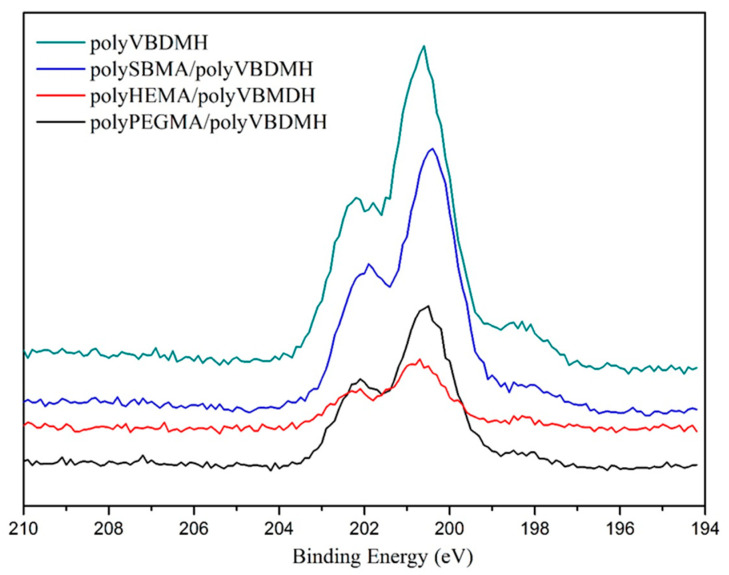
The XPS Cl 2p spectrum of different chlorinated VBDMH modified PU substrates.

The Cl2p was noted on all chlorinated VBDMH-modified PU substrates (Figure 5). Nevertheless, XPS is a surface-sensitive technique that only detects the chemical information on the top few hundred angstroms thick. Hence, the iodometric/thiosulfate titration method was utilized to determine the amount of oxidative chlorine (Cl^+^) on these VBDMH-modified PU substrates [22,23] The [Cl^+^] (μg/cm^2^) for these chlorinated specimens after 1 h of chlorination in a 10% diluted aqueous bleach solution were: polyPEGMA/polyVBDMH: 19.82 ± 1.18; polyHEMA/polyVBDMH: 8.28 ± 0.44; polySBMA/polyVBDMH: 13.90 ± 2.17; polyVBDMH: 10.06 ± 0.59. It was noted that the amount of Cl^+^ neither followed the order of surface hydrophilicity (Figure 2) nor the surface area percentage of –N–(C=O)–O (289.2 eV) that is related to the VBDMH chemical configuration (T 2). These implicated other factors could play roles in the chlorination reaction and further exploration was needed.

### 3.4. Antimicrobial Activity of Different Chlorinated VBDMH Modified PU Substrates

**Figure 6:**
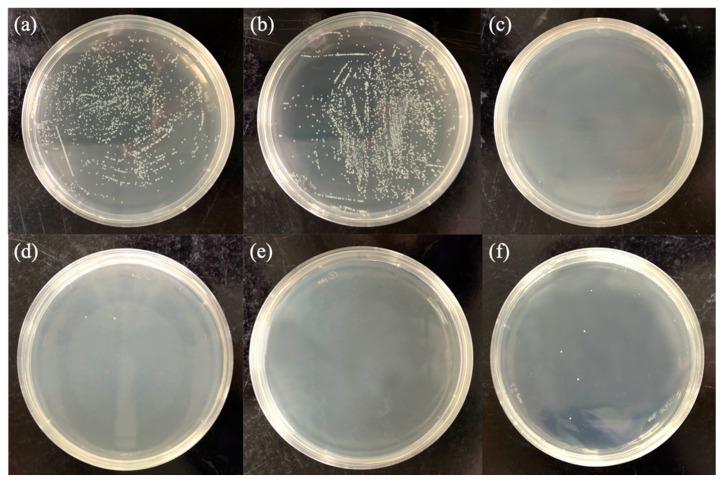
The representative agar plate image after antimicrobial assay against *E. coli* for (**a**) Pristable 0 h), (**b**) chlorinated (**c**) polyPEGMA/polyVBDMH, (**d**) polyHEMA/polyVBDMH, (**e**) polySBMA/VBDMH, and (**f**) polyVBDMH.

Figure 6 exhibits the representative agar plate images for the pristine PU incubated with *E. coli* for 0 h and 24 h, respectively, and different chlorinated VBDMH-containing PU substrates incubated with *E. coli* for 24 h. The highest number of colonies was noted on the pristine PU after 24 h of incubation. In contrast, nearly no colonies were noted on the chlorinated VBDMH-containing PU substrates, except for a few colonies noted on the PU substrate only modified with VBDMH (polyVBDMH). To further analyze the antimicrobial activity of these VBDMH-containing PU substrates, the lowest antimicrobial capability was found on the one only modified with a layer of VBDMH (i.e. polyVBDMH in Table 3). This could be attributed to the synergistic effects of the reduced chlorine content, as well as the reduced microbial contact due to the higher surface hydrophobicity associated with the polyVBDMH (Figure 2).

## 4. Conclusions

A polymerizable vinyl group-containing N-halamine, 3-(4’-vinylbenzyl)-5,5-dimethylhydantoin (VBDMH), was successfully synthesized and grafted onto polyurethane substrates via the surface-initiated atom transfer radical polymerization (SI-ATRP) technique with or without different prior SI-ATRP grafting of hydrophilic monomers. It was noted that the PU substrates with prior SI-ATRP grafting of hydrophilic monomers will turn hydrophobic after subsequent SI-ATRP of VBDMH monomers. Nevertheless, all these VBDMH-modified substrates with prior SI-ATRP grafting of hydrophilic monomers were more hydrophilic than those without. ATR-FTIR and XPS analyses indicated the PU surfaces were successfully modified by different SI-ATRP VBDMH modification schemes. Although the degree of chlorination of these different VBDMH-modified PU substrates cannot directly be predicted by the surface hydrophilicity nor by the surface chemical configuration, the lowest antimicrobial activity was noted on the one only modified with a layer of VBDMH, the most hydrophobic surface. This likely resulted from the synergistic effects caused by the reduced chlorine content and reduced contacts between the surface and microbes, *S. aureus*, or *E. coli*. This implicates, with proper prior chemical grafting of hydrophilic monomers, the antibacterial activity of the N-halamine-modified PU substrate can be improved. This could be of great use for preparing multifunctional substrates used in a wide variety of clinical applications.

## Figures and Tables

**Table 1:** The atomic percentage of different PU substrates.

	Atomic Percentage %					
	C1s	O1s	N1s	Br3d	S2p	O/C
Pristine PU	71.3	26.5	2.2	/	/	0.37
PU-OH	66.5	31.1	2.4	/	/	0.47
PU-Br	64.5	32.0	2.8	0.7	/	0.50
polyPEGMA	68.2	31.1	0.6	/	/	0.46
polyPEGMA/polyVBDMH	72.0	24.5	3.5	/	/	0.34
polyHEMA	66.7	32.3	0.9	/	/	0.48
polyHEMA/polyVBDMH	73.8	21.9	4.3	/	/	0.30
polySBMA	67.3	27.8	2.7	0.7	1.5	0.41
polySBMA/polyVBDMH	73.1	23.4	3.0	0.25	0.25	0.32
polyVBDMH	75.3	20.1	4.6	/	/	0.27

**Table 2:** The C1s curve fitting results of different PU substrates.

	Bond Percentage %			
	C–C 285.0 eV	C–O–C/C–O/C–N 286.5 eV	–O– (C=O) 288.9 eV	–N– (C=O) –O 289.2 eV
Pristine PU	69.4	16.8	6.5	7.2
PU-OH	62.8	21.8	6.5	8.9
PU-Br	61.6	28.6	6.7	3.0
polyPEGMA	37.4	55.8	6.3	0.6
polyPEGMA/polyVBDMH	54.7	35.2	/	10.1
polyHEMA	64.7	21.9	8.4	5.0
polyHEMA/polyVBDMH	65.8	20.8	3.0	10.4
polySBMA	63.3	26.2	7.5	3.0
polySBMA/polyVBDMH	60.7	25.9	3.1	10.3
polyVBDMH	63.9	21.9	/	14.3

**Table 3:** The antibacterial activity of different chlorinated VBDMH modified PU substrates. n = 3.

Specimen	Bacteria Log Reduction	
	*S. aureus*	*E. coli*
	(ATCC 21351)	(ATCC 23501)
polyPEGMA/polyVBDMH	2.44 ± 0.13	2.56 ± 0.00
polyHEMA/polyVBDMH	2.02 ± 0.17	2.02 ± 0.16
polySBMA/polyVBDMH	2.24 ± 0.20	2.40 ± 0.21
polyVBDMH	1.66 ± 0.08	1.63 ± 0.15

## Data Availability

The data presented in this study are available on request from the corresponding author.
